# Exploring bacterial interspecific interactions for discovery of novel antimicrobial compounds

**DOI:** 10.1111/1751-7915.12735

**Published:** 2017-05-29

**Authors:** Olaf Tyc, Victor C.L. de Jager, Marlies van den Berg, Saskia Gerards, Thierry K.S. Janssens, Niels Zaagman, Marco Kai, Ales Svatos, Hans Zweers, Cornelis Hordijk, Harrie Besselink, Wietse de Boer, Paolina Garbeva

**Affiliations:** ^1^ Department of Microbial EcologyNetherlands Institute of Ecology (NIOO‐KNAW)PO BOX 506700 ABWageningenThe Netherlands; ^2^ MicroLife Solutions B.V.Science Park 4061098 XHAmsterdamThe Netherlands; ^3^ Mass Spectrometry Research GroupMax Planck Institute for Chemical EcologyHans‐Knoell‐Str. 8D‐07745JenaGermany; ^4^ BioDetection Systems B.V.Science Park 4061098 XHAmsterdamThe Netherlands; ^5^ Department of Soil QualityWageningen University and Research Centre (WUR)PO BOX 476700 AAWageningenThe Netherlands

## Abstract

Recent studies indicated that the production of secondary metabolites by soil bacteria can be triggered by interspecific interactions. However, little is known to date about interspecific interactions between Gram‐positive and Gram‐negative bacteria. In this study, we aimed to understand how the interspecific interaction between the Gram‐positive *Paenibacillus* sp. AD87 and the Gram‐negative *Burkholderia* sp. AD24 affects the fitness, gene expression and the production of soluble and volatile secondary metabolites of both bacteria. To obtain better insight into this interaction, transcriptome and metabolome analyses were performed. Our results revealed that the interaction between the two bacteria affected their fitness, gene expression and the production of secondary metabolites. During interaction, the growth of *Paenibacillus* was not affected, whereas the growth of *Burkholderia* was inhibited at 48 and 72 h. Transcriptome analysis revealed that the interaction between *Burkholderia* and *Paenibacillus* caused significant transcriptional changes in both bacteria as compared to the monocultures. The metabolomic analysis revealed that the interaction increased the production of specific volatile and soluble antimicrobial compounds such as 2,5‐bis(1‐methylethyl)‐pyrazine and an unknown Pederin‐like compound. The pyrazine volatile compound produced by *Paenibacillus* was subjected to bioassays and showed strong inhibitory activity against *Burkholderia* and a range of plant and human pathogens. Moreover, strong additive antimicrobial effects were observed when soluble extracts from the interacting bacteria were combined with the pure 2,5‐bis(1‐methylethyl)‐pyrazine. The results obtained in this study highlight the importance to explore bacterial interspecific interactions to discover novel secondary metabolites and to perform simultaneously metabolomics of both, soluble and volatile compounds.

## Introduction

Recent studies have shown that interspecific interactions between soil bacteria can strongly effect their behaviour and the secretion of secondary metabolites (Seyedsayamdost *et al*., 2012; Traxler *et al*., 2013; Tyc *et al*., 2014). The soil and rhizosphere are characterized by high complexity, diversity and density of microorganisms (Gans *et al*., 2005; Uroz *et al*., 2010). In these environments, microorganisms interact in different ways ranging from competition to cooperation (Czaran and Hoekstra, 2009; Foster and Bell, 2012; Allen and Nowak, 2013). Many soil bacterial species have overlapping metabolic niches and use similar substrates as energy source for their growth and persistence (Yin *et al*., 2000; Demoling *et al*., 2007; Strickland *et al*., 2009). Consequently, competition for nutrients is one of the most abundant forms of interaction occurring in soil and rhizosphere bacterial communities (Demoling *et al*., 2007; Rousk *et al*., 2009). To sustain in such demanding environmental conditions bacteria evolved the ability to produce and secrete secondary metabolites with antimicrobial properties (e.g. antibiotics, siderophores, bacteriocins, volatiles and others) as competitive tools for their survival (Hibbing *et al*., 2010). Comprehensive knowledge of bacterial interspecific interactions is important for better understanding of soil microbial community composition and soil functions such as disease suppression and plant growth promotion.

Previously, we have performed a high‐throughput screening of interaction‐mediated induction of antimicrobial compound production by bacterial strains obtained from the rhizosphere and bulk soil of *Carex arenaria* stands (Tyc *et al*., 2014). A clear case of such interaction‐mediated triggering of antimicrobial activity was observed when a *Burkholderia* and a *Paenibacillus* strains were co‐cultured. So far, very little is known about the interactions between Gram‐positive and Gram‐negative bacteria and the triggering of secondary metabolite production during such interactions.

Bacteria belonging to the genus *Burkholderia* are Gram‐negative, non‐spore forming *Proteobacteria*. They occupy diverse range of ecological niches (van Elsas *et al*., 2002; Salles *et al*., 2002; Coenye and Vandamme, 2003; Compant *et al*., 2008) and their lifestyle can range from free living in soil and rhizosphere to endo‐ and epiphytic, including obligate endosymbionts and plant pathogens (Coenye and Vandamme, 2003; Compant *et al*., 2005; Vial *et al*., 2011). In recent years, the interest on *Burkholderia* strains has increased as these bacteria have shown to have compelling properties for agriculture like plant growth promotion, increasing in diseases resistance, improvement of nitrogen fixation and phosphorus utilization (Nowak and Shulaev, 2003; Sessitsch *et al*., 2005; Schmidt *et al*., 2009; Groenhagen *et al*., 2013; Zhao *et al*., 2014).

Soil bacteria belonging to the genus *Paenibacillus* are Gram‐positive, facultative anaerobe and endo‐spore forming bacteria (von der Weid *et al*., 2000; da Mota *et al*., 2005). Bacteria of this genus are able to colonize diverse habitats like water, soil and insects (Berge *et al*., 2002; Bosshard *et al*., 2002; Daane *et al*., 2002; Peters *et al*., 2006; Timmusk *et al*., 2009). Many studies have shown that paenibacilli play an important role as plant growth‐promoting rhizobacteria (PGPR) for plant health and growth (e.g. nitrogen fixation and pest control) (McSpadden Gardener, 2004; Ryu *et al*., 2005; Anand *et al*., 2013; Debois *et al*., 2013). Furthermore members of the genus *Paenibacillus* are known as a rich source for chemical compounds useful in the field of biotechnology and agriculture such as antibiotics, enzymes and other bioactive molecules (Wu *et al*., 2010; Debois *et al*., 2013; Cochrane and Vederas, 2016).

The main goal of this study was to obtain insight into the interspecific interaction between *Burkholderia* sp. AD24 and *Paenibacillus* sp. AD87 using metabolomic and transcriptome techniques.

## Results

### Effect of interspecific interaction on *Burkholderia* sp. AD24 and *Paenibacillus* sp. AD87 cell numbers

**Figure 1:**
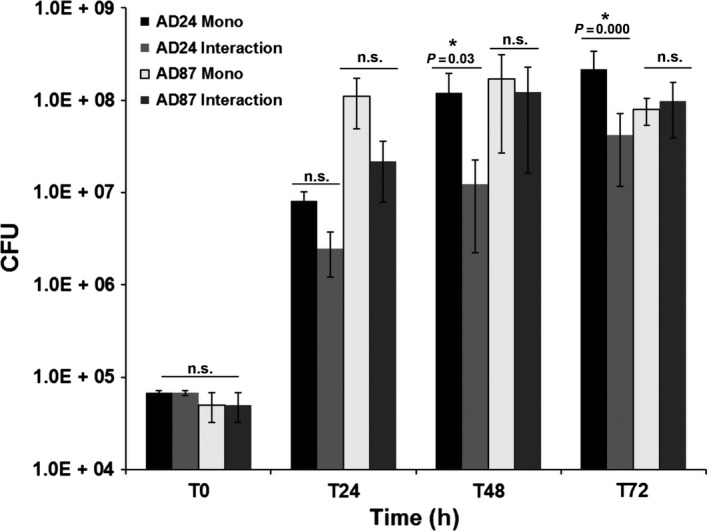
Average colony‐forming units (CFU) revealed during the interaction between *Burkholderia* sp. AD24 and *Paenibacillus* sp. AD87 grown on 1/10th TSBA plates. Significant differences between treatments (pairwise combinations) and the control (monocultures) are indicated by asterisks (one‐way ANOVA,* post hoc* TUKEY test *P *<* *0.05). Abbreviations: AD24 Mono: *Burkholderia* sp. AD24 monoculture, AD24 Interaction: *Burkholderia* sp. AD24 in interaction with *Paenibacillus* sp. AD87. AD87 Mono: *Paenibacillus* sp. AD87 monoculture, AD87 interaction: *Paenibacillus* sp. AD87 in interaction with *Burkholderia* sp. AD24.

Bacterial colony‐forming units obtained from monocultures and interactions are summarized in Fig. 1. *Burkholderia* reached the highest density in monoculture after 72 h of incubation (2.17 × 10^8^ CFU). The growth of *Burkholderia* was negatively affected when confronted with the Gram‐positive *Paenibacillus* strain resulting in significantly lower cell counts compared to the monoculture at 48 and 72 h of incubation (Fig. 1).

In monocultures, *Paenibacillus* reached 1.69 × 10^8^ CFU after 48 h and 7.92 × 10^7^ CFU after 72 h of incubation. During interaction, the growth of *Paenibacillus* was not significant affected when confronted with *Burkholderia* (Fig. 1). Overall the results revealed that the growth of *Burkholderia* was more negatively affected during the interaction as compared to the growth of *Paenibacillus*.

### Genomic features of *Burkholderia* sp. AD24 and *Paenibacillus* sp. AD87

The genome of *Burkholderia* consisted of two chromosomes and one plasmid with a total size of 8.2 MB. The genome of *Paenibacillus* consisted of one chromosome with a size of 7.1 MB Table S1. The antiSMASH *in silico* secondary metabolite analysis on the genome of *Burkholderia* revealed a total of 14 gene clusters from which five gene clusters belonged to the class of bacteriocins, three to the class of terpenes, two to non‐ribosomal peptides, two to NRPS‐Hser‐lactones, one to the class of type‐3 polyketide synthase and one to the class of phosphonates. For *Paenibacillus,* the *in silico* analysis revealed a total 10 gene clusters from which two gene clusters belonged to the class of Terpenes, one to bacteriocins, one to lassopeptides, two to the class of lantipeptides, one to non‐ribosomal peptides, one to others, one to the class of type‐3 polyketide synthase and one to the class of siderophores.

### Effect of interspecific interactions on gene expression

**Figure 2:**
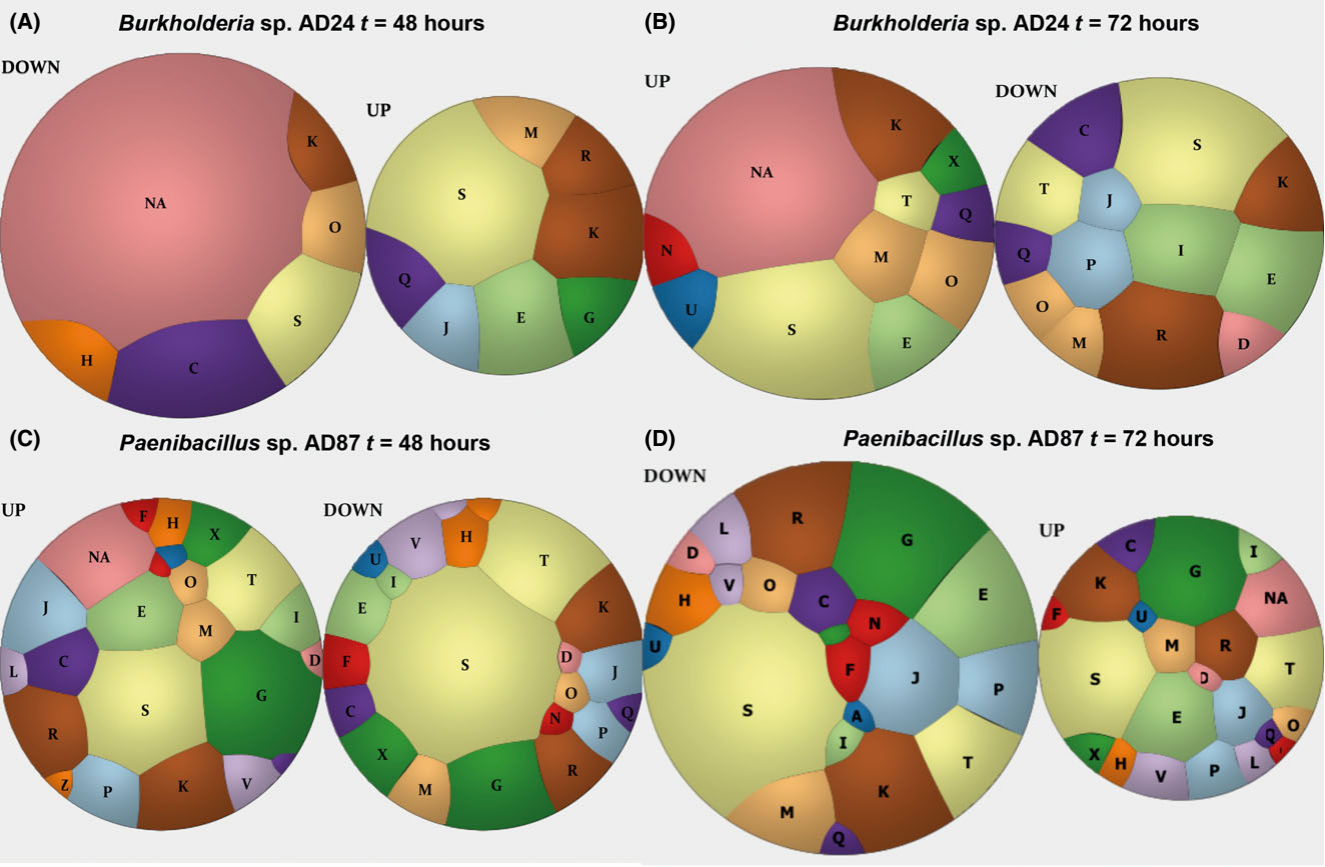
Overview of the transcriptome analysis for *Burkholderia* sp. AD24 and *Paenibacillus* sp. AD87 during interaction. A. Classification of significantly differentially expressed genes in *Burkholderia* sp. AD24 during interspecific interaction with *Paenibacillus* sp. AD87 at 48 h of incubation. B. Differentially expressed genes based on COG classification for *Burkholderia* sp. AD24 in interaction with *Paenibacillus* sp. AD87 at 72 h of incubation. C. Differentially expressed genes based on COG classification for *Paenibacillus* sp. AD87 in interaction with *Burkholderia* sp. AD24 at 48 h of incubation. D. Differentially expressed genes based on COG classification for *Paenibacillus* sp. AD87 in interaction with *Burkholderia* sp. AD24 at 72 h of incubation. The one‐letter codes represent the following functional categories: A: RNA processing and modification, B: chromatin structure and dynamics, C: energy production and conversion; D: cell cycle control, cell division, chromosome partitioning; E: amino acid transport and metabolism; F: nucleotide transport and metabolism; G: carbohydrate transport and metabolism; H: coenzyme transport and metabolism; I: lipid transport and metabolism; J: translation, ribosomal structure and biogenesis; K: transcription; L: replication, recombination and repair; M: cell wall/membrane/envelope biogenesis; N: cell motility; NA: not assigned; O: post‐translational modification, protein turnover; chaperones; P: inorganic ion transport and metabolism; Q: secondary metabolites biosynthesis, transport and catabolism; R: general function prediction only; S: function unknown; T: signal transduction mechanisms; U: intracellular trafficking, secretion, and vesicular transport; V: defence mechanisms X: mobilome: prophages, transposons. The circle size is scaled to the number of differentially expressed genes of each COG category at each time point.

Transcriptome analysis revealed that the interaction between *Burkholderia* and *Paenibacillus* caused transcriptional changes in both bacteria as compared to the monocultures. At 48 h of incubation, the expression of 38 genes (14 up‐ and 24 downregulated) was significantly affected in *Burkholderia* whereas 531 genes were significantly differentially expressed in *Paenibacillus* (310 up‐ and 221 downregulated) (Fig. 2A,C Tables S2 and S3). The highest number of differentially expressed genes was observed at 72 h of incubation with 62 genes differentially expressed in *Burkholderia* (33 up‐ and 29 downregulated) and 1114 genes in *Paenibacillus* (381 up‐ and 733 downregulated) (Fig. 2B,D, Tables S2 and S3). Analysis based on orthologous gene categories (COG) revealed that most of the up‐ and downregulated genes in *Burkholderia* belonged to the categories C (energy production and conversion), E (amino acid transport and metabolism), G (carbohydrate transport and metabolism), NA (not assigned), S (function unknown), I (Lipid transport/metabolism) and K (transcription) (Fig. 2A,B). For *Paenibacillus,* most of the differentially expressed genes belonged to the categories G (carbohydrate transport/metabolism), K (transcription), R (general function prediction only), S (function unknown), NA (not assigned) and T (signal transduction mechanisms) (Fig. 2C,D).

### Differentially expressed genes related to secondary metabolite production

In *Burkholderia,* the gene bAD24_II06980 (carboxymethylenebutenolidase) was differentially expressed at 48 and 72 h and 2.27‐fold upregulated as compared to the monoculture (Table S2). In *Paenibacillus* at 72 h of incubation, a total of three genes related to secondary metabolite production were highly expressed namely: gpAD87_13790 (Acetyl esterase Axe7A), gpAD87_02615 (imidazolonepropionase) and gene gpAD87_01890 (homoserine O‐acetyltransferase). These genes were respectively 3.49‐fold and 1.9‐fold higher expressed during interaction with *Burkholderia* (Table S2).

### Differentially expressed genes related to signal transduction

During the interaction between *Burkholderia* and *Paenibacillus,* several genes related to the signal transduction systems (category T) were affected. In *Burkholderia,* the gene bAD24_p01570 (Cyclic di‐GMP phosphodiesterase response regulator RpfG) was 2.15‐fold higher expressed as compared to the monoculture (Table S2). Interestingly, this gene was found on a mobile genetic element. In *Paenibacillus,* 57 genes related to signal transduction were affected. The genes gpAD87_21325 (Transcriptional regulatory protein LiaR) and gpAD87_11325 (Sensor histidine kinase YehU) were the most affected with 6.84‐ up‐ and 7.91‐fold change downregulated respectively (Table S3). At 72 h, 30 genes were upregulated and 41 genes were downregulated (Fig. 2C) from which gene gpAD87_26840 (Methyl‐accepting chemotaxis protein McpB) and gpAD87_07465 (Low‐molecular weight protein‐tyrosine‐phosphatase YfkJ) were the most affected with 7.41 up‐ and 8.46 downregulated respectively (Table S3).

### Differentially expressed genes related to defence mechanisms

In total, 22 genes belonging to defence mechanisms were affected in *Paenibacillus* after 48 h (eight upregulated, 14 downregulated) and 19 genes were affected at 72 h (12 upregulated and seven downregulated) (Fig. 2C,D). At both time points, the most affected genes in *Paenibacillus* were gpAD87_28110 (Vancomycin B‐type resistance protein (VanW) and gpAD87_18840 (putative ABC transporter permease) (Table S3). The two most downregulated genes at 48 h were the genes gpAD87_12115 (Multidrug resistance protein (YkkD)) and gpAD87_09055 (Multidrug resistance protein (NorM)) with 6.73‐ and 4.67‐fold changes respectively (Table S3). At 72 h, the genes gpAD87_14640 (Putative penicillin‐binding protein (Pbpx)) and gpAD87_06980 (RutC family protein) were downregulated with fold changes of 6.94 and 5.96 respectively (Table S3).

### Effect of interspecific interaction on secondary metabolite production

#### Soluble metabolites

**Figure 3:**
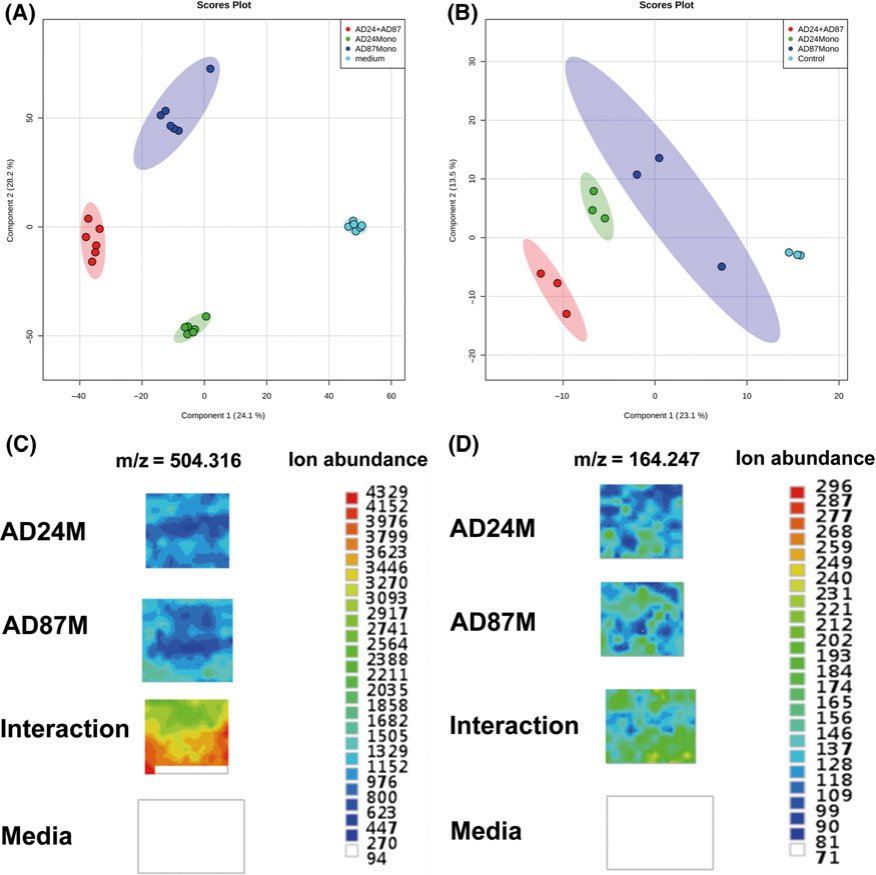
Metabolomic analysis of monocultures and interactions of *Burkholderia* sp. AD24 and *Paenibacillus* sp. AD87 (A) PLSDA 2D‐plot of the analysed LC‐MS data of soluble compounds after three days of incubation. (B) PLSDA 2D‐plot of GC‐MS data of volatiles emitted after three days of inoculation. (C) Results of the LAESI‐MS imaging: heat map targeting the Pederin‐like compound with an *m/z* of 504.316 [M+H+] showing specific accumulation of ions related to this compound in the interaction of *Paenibacillus* sp. AD87 (AD87M) and *Burkholderia* sp. AD24 (AD24M) (D) Results of the LAESI‐MS imaging: heat map targeting 2,5‐bis(1‐methylethyl)‐pyrazine with an *m/z* of 164.247 showing specific accumulation of ions related to this compound in interaction samples of *Paenibacillus* sp. AD87 with *Burkholderia* sp. AD24 (Interaction). The colour associated with the ion map represents the base peak intensity (BPI) of [M+H+] masses at a 1–2 ppm window, scale bar of ion abundance on the right side.

Metabolome analysis performed on secondary metabolite extracts of monocultures, and interactions revealed that the metabolites composition of the monocultures differed from that of the mixtures (Fig. 3A). Clear separations of metabolite composition between controls, monocultures and interactions were obtained in partial least squares discriminant analysis (PLSDA) score plots (Fig. 3A). One of the compounds observed in a higher concentration during interaction was identified as an unknown compound with the same molecular mass as Pederin (C_25_H_45_NO_9_, *m/z *=* *504.316) [M+H^+^] (Fig. S1).

#### Volatile metabolites

The comparison of volatile organic compounds emitted by the two bacteria revealed clear separations between the monocultures, controls and the interaction based on partial least squares discriminant analysis (PLSDA) (Fig. 3B). The analysis revealed 19 volatile organic compounds produced by bacteria that were not detected in the controls (Table 1). In total, 14 volatile organic compounds could be tentatively identified and categorized in six different chemical classes (alkenes, benzoids, sulfides, terpenes, furans and pyrazines). Five compounds could not be assigned with certainty and remained unknown. The most prominent headspace volatile organic compounds were the two sulfur‐containing compounds dimethyl disulfide (C_2_H_6_S_2_) and dimethyl trisulfide (C_2_H_6_S_3_), which were produced by both *Burkholderia* and *Paenibacillus*. Interestingly two volatile compounds produced by the monoculture of *Burkholderia* (S‐Methyl methanethiosulfonate and unknown compound 4) were not detected during the interaction with *Paenibacillus* (Table 1). One volatile organic compound was produced in higher abundance during the interaction. This compound was identified as 2,5‐bis(1‐methylethyl)‐pyrazine (C_10_H_16_N_2_, *m/z *=* *164.247, RT = 19.7) (Fig. S2, Table 1). For compound confirmation and bioassays, 2,5‐bis(1‐methylethyl)‐pyrazine was commercially synthesized and GC/MS spectra obtained from the samples were compared to the pure compound.

**Table 1:** Tentatively identified volatile organic compounds (VOCs) produced by *Burkholderia* sp. AD24 and *Paenibacillus* sp. AD87 in mono‐ and co‐culture on 1/10th TSB agar

#	Compound name	RT*	ELRI*	*P*‐value***	Chemical class	Detected in treatment		
						Burk	Paen	MIX Burk+Paen
1	1,3‐butadiene, 2‐methyl‐	2.11	525	0.018	Alkenes	X	X	X
2	2‐methylfuran	2.53	586	0.030	Furan	X	X	X
3	Dimethyl disulfide	4.20	741	0.001	Sulfides	X	X	X
4	Toluene	4.63	762	0.000	Benzenoids	X	X	X
5	Unknown compound 1	5.25	786	0.000	–	X	X	X
6	1,3‐dithiethane	5.44	793	0.001	Sulfides	X		X
7	2,4 dithiapentane	7.96	887	0.009	Sulfides	X		X
8	Alpha‐pinene	9.59	930	0.011	Terpenes	X	X	X
9	Benzaldehyde	10.53	956	0.016	Aldehydes	X	X	X
10	Unknown compound 2	10.63	959	0.017	–	X	X	X
11	Dimethyl trisulfide	10.86	964	0.027	Sulfides	X	X	X
12	Unknown alkene	12.41	1003	0.018	Alkenes	X	X	X
13	Unknown compound 3	13.87	1040	0.013	–	X	X	X
14	S‐Methyl methanethiosulfonate	14.65	1059	0.010	Sulfides	X		
15	1,2,4‐Trithiolane	15.71	1082	0.012	Sulfides	X		X
16	Unknown compound 4	15.89	1087	0.000	–	X	X	
17	2,5‐bis(1‐methylethyl)‐pyrazine	19.73	1186	0.001	Pyrazines		X	X
18	Branched alkene	23.19	1284	0.008	Alkenes	X	X	X
19	Unknown compound 5	30.81	1471	0.008	–	X	X	X
Number of detected compounds (*n*)						20	16	19

# = Compound number, Burk = *Burkholderia* sp., AD24, Paen = *Paenibacillus* sp. AD87, MIX Burk+Paen = *Burkholderia* sp., AD24 +  *Paenibacillus* sp. AD87, RT* = Retention time, the RT value stated is the average of three replicates, ELRI** = Experimental linear retention index value, the RI value stated is the average of three replicates.

***P‐value = statistical significance (peak area and peak intensity).

### LAESI‐mass spectrometry (ambient imaging mass spectrometry)

With the LAESI‐mass spectrometry system, we confirmed the production of the two identified compounds 2,5‐bis(1‐methylethyl)‐pyrazine (*m/z *=* *164.247) and the unknown Pederin‐like compound (*m/z *=* *504.316) [M+H^+^]) directly on living microcolonies of *Burkholderia* and *Paenibacillus*, without extraction (Fig. 3C,D).

### Biological activity of 2,5‐bis(1‐methylethyl)‐pyrazine against *Burkholderia* sp. AD24 and *Paenibacillus* sp. AD87

**Figure 4:**
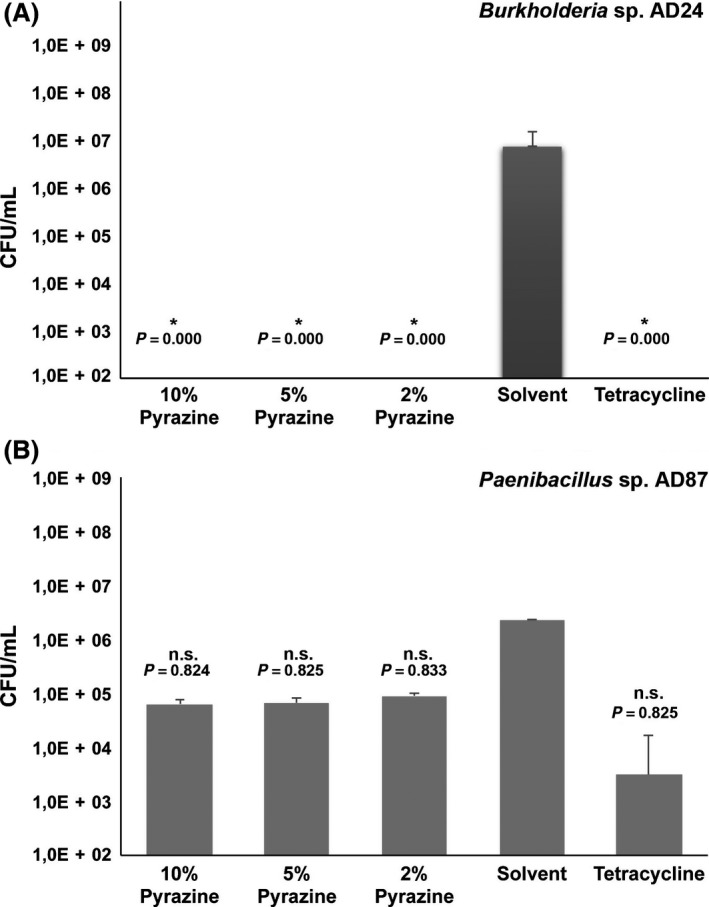
Effect of 2,5‐bis(1‐methylethyl)‐pyrazine on bacterial growth of 10%, 5% and 2% v/v 2,5‐bis(1‐methylethyl)‐pyrazine. A. *Burkholderia* sp. AD24 compared to the controls: solvent and the tetracycline. B. *Paenibacillus* sp. AD87 compared to the controls: solvent and the tetracycline. Bars represent the average number of obtained colony‐forming units per ml (CFU ml^−1^). Error bars indicate the standard deviation (SD) between the replicates. Asterisks indicate significant differences between the treatments and the solvent control (one‐way ANOVA *post hoc* TUKEY test *P *<* *0.05).

The *in vitro* test with 2,5‐bis(1‐methylethyl)‐pyrazine revealed a significant growth inhibition on the cell counts of *Burkholderia* (Fig. 4A). The growth of *Burkholderia* was significantly (*P *=* *0.000) inhibited by all applied concentrations 10%, 5% and 2% v/v compared to the control (*Burkholderia* grown in absence of the pyrazine compound) were *Burkholderia* reached a cell density of 7.15 × 10^6^ cells ml^−1^. The growth of *Paenibacillus* was not significantly inhibited (*P *=* *0.824, *P *=* *0.825 and *P *=* *0.833) by the application of 10%, 5% and 2% v/v of 2,5‐bis(1‐methylethyl)‐pyrazine (Fig. 4B).

### Biological activity of 2,5‐bis(1‐methylethyl)‐pyrazine against fungi and human pathogenic model organisms in agar diffusion assays

After overnight incubation, significant growth inhibition of *E. coli* WA321, *S. aureus* 533R4 and *C. albicans* BSMY212 was observed by exposure to 1.84 mg of pure 2,5‐bis(1‐methylethyl)‐pyrazine (Fig. S3A). Significant growth inhibition on the plant pathogenic fungi *R. solani* AG2.2IIIB and *F. culmorum* PV was also observed by application of 1.84 mg 2,5‐bis(1‐methylethyl)‐pyrazine (Fig. S3B).

### Antibacterial activity of soluble secondary metabolites

Agar diffusion tests performed with secondary metabolite extracts obtained from monocultures and interactions of *Burkholderia*,* Paenibacillus* revealed antimicrobial activity against *E. coli* WA321 (ig. FS3C) and *S. aureus* 533R4 (Fig. S4). The growth was significantly inhibited, however without difference in inhibition between extracts obtained from monocultures or interactions (Figs S3C and S4).

### Additive effect of diffusible and volatile secondary metabolites

Agar diffusion tests performed with secondary metabolite extracts from the interacting bacteria, in combination with the pure volatile compound 2,5‐bis(1‐methylethyl)‐pyrazine revealed additive effects between soluble and volatile compounds against *E. coli* WA321. The exposure to the secondary metabolite extract in combination with pure 2,5‐bis(1‐methylethyl)‐pyrazine led to significant bigger zones of inhibition (ZOI) compared to the controls (secondary metabolite extracts without added pure volatile compound) (Fig. S3D). There was no significant additive effect against the Gram‐positive model organism *S. aureus* 533R4 (Fig. S4).

## Discussion

Phenotypic changes occurring during microbial interspecific interactions are receiving increased attention, as they are the basis for understanding microbial communities (Seyedsayamdost *et al*., 2012; Traxler *et al*., 2013). Interspecific interactions between soil bacteria were shown to have a major impact on production of antimicrobial compounds, with both induction and suppression of antimicrobial activity (Tyc *et al*., 2014).

In this study, we revealed that the interaction between *Burkholderia* and *Paenibacillus* had a significant negative effect on *Burkholderia* cell numbers at 48 and 72 h whereas cell numbers of *Paenibacillus* were not significantly affected during the interaction. Hence, *Paenibacillus* is a better competitor than *Burkholderia* under the tested conditions. Similar observations were previously reported for the interspecific interaction between the Gram‐negative *Pseudomonas fluorescens* Pf0‐1 and the Gram‐positive *Bacillus* sp. V102 (Garbeva *et al*., 2011; Tyc *et al*., 2015).

The transcriptome analysis revealed that both bacteria responded with changes in gene expression with higher number of significantly differentially expressed genes in *Paenibacillus*. However, several of the differentially expressed genes in *Burkholderia* and *Paenibacillus* strains are hypothetical proteins. Despite the advantages made in the field of genome sequencing and annotation, a vast percentage of bacterial genome sequences (~40%) remain with unknown function (Galperin and Koonin, 2004; Song *et al*., 2015).

In *Burkholderia*, we observed differential expression of ribosomal proteins that may point to a general stress response as ribosomal proteins may have various functions apart from protein synthesis (Ishige *et al*., 2003; Silberbach and Burkovski, 2006; Picard *et al*., 2013) and can be important for antimicrobial activity (de Carvalho *et al*., 2010).

Upregulation of several genes related to signal transduction, secondary metabolite production and to cell motility was observed for *Burkholderia* during the interaction with *Paenibacillus*. The elevated expression of gene bAD24_II08070 YiaD which is associated with the flagellar biogenesis and the cellular motility apparatus (Hu *et al*., 2009) indicates that motility may be an important escape strategy during bacterial interspecific interactions (Garbeva *et al*., 2011, 2014b). Interestingly, the highest fold change in gene expression in *Burkholderia* was found for the gene bAD24_p01665, which is related to the type IV secretion system. This secretion system plays an important role for the virulence of *Burkholderia* spp. (Zhang *et al*., 2009). The gene encoding for this secretion system was found on the mobile genetic element*,* which is in line with previous reports (Engledow *et al*., 2004).

Genes encoding for antibiotic resistance was highly upregulated in *Paenibacillus*. In particular, gene gpAD87_28110 encoding the Vancomycin B‐type resistance gene VanW was 9.36‐fold upregulated, suggesting protection against antimicrobial compounds produced during the interaction. So far, the exact function of the gene VanW is unknown (Evers and Courvalin, 1996; McGregor and Young, 2000).

The metabolomics analysis revealed that the interspecific interaction between *Burkholderia* and *Paenibacillus* increased the production of antimicrobial compounds such as 2,5‐bis(1‐methylethyl)‐pyrazine and an unknown compound with the same molecular mass as Pederin. These two compounds were detected in higher concentrations during interspecific interaction using three independent approaches namely Orbitrap‐XL‐MS, GC/MS‐Q‐TOF and ambient imaging mass spectrometry (LAESI‐ MS) from living bacterial colonies.

The growth of *Burkholderia* was significantly inhibited by 2,5‐bis(1‐methylethyl)‐pyrazine, which indicates that *Paenibacillus* is the producer of the identified pyrazine compound. Additional 2,5‐bis(1‐methylethyl)‐pyrazine revealed significant antibacterial and antifungal activity against a range of human and plant pathogenic model organisms. This is in line with previous studies revealing that pyrazine compounds exhibit antimicrobial activities (Beck *et al*., 2003; Kucerova‐Chlupacova *et al*., 2015). The bacterial production of 2,5‐bis(1‐methylethyl)‐pyrazine was so far only reported for few bacteria including *Paenibacillus* (Beck *et al*., 2003; Dickschat *et al*., 2005; Rajini *et al*., 2011). Another compound produced in higher concentration during the interspecific interaction of *Burkholderia* and *Paenibacillus* was a soluble compound with an *m/z* of 504.316 [M+H^+^]. This compound was identified as a Pederin‐like compound (exact mass difference < 0.5 ppm); however, analysis of *Paenibacillus* genome data revealed that not all gene clusters needed for Pederin production are present in the genome of *Paenibacillus*. This suggests that the detected compound might be a novel compound with the same molecular mass as Pederin.

Increased antimicrobial effects were seen when the extracted soluble compounds and the pure volatile compound 2,5‐bis(1‐methylethyl)‐pyrazine were combined (Fig. S3D). This might be due to synergistic or additive effects between soluble and volatile compounds enhancing the overall antimicrobial activity (Schmidt *et al*., 2015). Such additive effects between volatile and soluble compounds were previously observed for hydrophilic antibiotics like beta‐lactams, which showed only negligible antimicrobial effects on Gram‐negative bacteria if applied alone (Hemaiswarya and Doble, 2010). However, if these antibiotics were applied together with the volatile eugenol, increased antimicrobial activity towards Gram‐negative and Gram‐ positive bacteria was observed (Hemaiswarya and Doble, 2010). In addition, combinations of soluble and volatile compounds have shown to inhibit the growth of multiresistant *E. coli* and *S. aureus* (Gallucci *et al*., 2009).

The production of secondary metabolites with antimicrobial properties such as 2,5‐bis(1‐methylethyl)‐pyrazine may offer competitive advantages during interspecific interactions for the producing strain by inhibition of surrounding competitors. Indeed, our results revealed that *Paenibacillus* performed better during the interaction by induction of motility in *Burkholderia* and reducing its growth.

Our present study on interspecific bacterial interactions has been performed on nutrient rich agar media (1/10th TSBA); these growth conditions are different from the nutritional conditions in natural soils (Torsvik *et al*., 1990; Demoling *et al*., 2007). However, in a soil‐microcosm performed with a synthetic microbial community consisting out of five bacterial species, including *Burkholderia* sp. AD24 and *Paenibacillus* sp. AD87, the 2,5‐bis(1‐methylethyl)‐pyrazine was also produced (Schulz‐Bohm *et al*., 2015). Thus, it is very likely that this pyrazine compound will be produced in natural systems such as soil.

In conclusion, the present study revealed that interspecific bacterial interactions affected fitness, gene expression and the production of volatile and soluble secondary metabolites. The results obtained in this study highlight the importance to explore bacterial interspecific interactions for discovery of novel secondary metabolites and the importance to perform simultaneously metabolomics of both, soluble and volatile compounds, which may have additive or synergistic effects.

## Experimental procedures

### Bacteria and culture conditions

Based on a previous high‐throughput screening on 146 soil isolates (Tyc *et al*., 2014), a Gram‐negative strain, *Burkholderia* sp. AD24 (*Beta‐proteobacteria*) and a Gram‐positive strain, *Paenibacillus* sp. AD87 (*Firmicutes*) were selected for this study. The bacterial isolates were pre‐cultured from −80°C glycerol stocks on 1/10th TSBA (Garbeva and de Boer, 2009) for three days at 24°C. Two indicator bacteria, *Escherichia coli* WA321 and *Staphylococcus aureus* 533R4 were used as target bacteria to detect the production of antimicrobial compounds (Meyer and Schleifer, 1978; Tyc *et al*., 2014). The indicator bacteria were pre‐cultured from −80°C glycerol stocks on LB‐A media (LB‐Medium Lennox, Carl Roth GmbH + Co. KG, 20 g l^−1^ Merck Agar). The indicator bacteria were incubated overnight at 37°C prior application. All bacterial isolates are listed in Table S4.

### Eukaryotic model organisms and culture conditions

The plant pathogens *Rhizoctonia solani* AG2.2IIIB and *Fusarium culmorum* PV were used as fungal model organisms (Garbeva *et al*., 2014a). The fungi were pre‐cultured on 1/5th potato dextrose agar (PDA) (29 g l^−1^ Oxoid CM 139) (Fiddaman and Rossall, 1993) and incubated at 24°C for 7 days. As model organism for yeast‐like fungi, *Candida albicans* BSMY 212 (DSMZ # 10697) was used. *C. albicans* BSMY 212 (Schmidt, 1996) was pre‐cultured from −80°C glycerol stocks on YEPD plates (20 g l^−1^ Merck Dextrose, 20.0 g l^−1^ BACTO™ Peptone, 10.0 g l^−1^ BACTO™ Yeast extract, 20 g l^−1^ Merck Agar). All eukaryotic model organisms are listed in Table S4.

### Bacterial interaction assay

One single colony of each bacterial isolate was picked and inoculated in 20 ml 1/10th TSB and grown overnight at 24°C, 220 rpm to an optical density of OD_600_ 0.630 (*Burkholderia*) and OD_600_ 0.680 (*Paenibacillus*). An inoculation mix of each treatment (*Burkholderia* monoculture, *Paenibacillus* monoculture and the mix of both) was prepared by diluting the bacterial isolates in 20 ml of 10 mM phosphate buffer (pH 6.5) to an OD_600_ of 0.002 (*Burkholderia*) and 0.005 (*Paenibacillus*) corresponding to ~5 × 10^5^ CFU ml^−1^ (in monoculture and interaction). Each inoculation mix was pulse‐vortexed for 30 s, and a volume of 100 μl was spread with a drigalski spatula in triplicates on 1/10th TSBA plates. Plates were incubated at 24°C, and sampling for bacterial cell counts, total RNA and secondary metabolite extraction was performed at 24, 48 and 72 h of incubation.

### Enumeration of bacterial growth

Bacterial growth was determined by selective plate counting. After 24, 48 and 72 h of incubation, a volume of 1 ml of 10 mM phosphate buffer (pH 6.5) was added to the surface of the 1/10th TSBA plates and all grown colonies were suspended from the plate surface using a disposable cell scratcher. The resulting cell suspension was transferred to a 15 ml Greiner tube containing 9 ml of 10 mM phosphate buffer (pH 6.5) and homogenized by vortex for 30 s. Dilution series of each treatment were prepared in triplicates. A volume of 100 μl of each serial dilution was plated in three replicates on 1/10th TSBA plates supplemented with either streptomycin 25 μg ml^−1^ (*Paenibacillus*) or vancomycin 50 μg ml^−1^ (*Burkholderia*). The plates were incubated for three days at 20°C, and bacterial enumeration was carried out on an aCOlyte Colony Counter (Don Whitley Scientific).

### RNA sampling and isolation

For RNA extraction, 0.5 ml of each sample replicate and at each time point (monocultures and interaction) was transferred to a 2 ml tube containing 1 ml RNA protect Bacteria Reagent (Qiagen, cat# 76506, Qiagen Benelux B.V., Venlo, The Netherlands) and centrifuged for 10 min at 10 000 × *g*, and 4°C. The supernatant was discarded, and cells were directly frozen in liquid N_2_ and immediately stored at −80°C. Total RNA was extracted with the Aurum Total RNA Mini Kit (BIO‐ RAD cat# 732‐6820) according to the manufacturer's protocol. After extraction samples were treated with the TURBO DNA‐free Kit (AMBION cat# 1907, Life Technologies Europe B.V., Bleiswijk , The Netherlands). The RNA concentration and quality was checked on a NanoDrop Spectrophotometer and on a 1.0% agarose gel. Samples were subjected to RNA sequencing at Baseclear using the Illumina Sequencing platform.

### Whole genome DNA isolation and genome sequencing

Genomic DNA of *Burkholderia* and *Paenibacillus* was extracted from overnight cultures using the QIAGEN genomic DNA Mini Kit (Qiagen, cat# 13323) (for Illumina Sequencing) or using the QIAGEN MagAttract HMW DNA Kit (Qiagen, cat# 67563) from exponentially growing overnight cultures for PacBio sequencing. The extracted DNA was dissolved in 100 μl sterile nuclease‐free water and quantified with a NanoDrop Spectrophotometer. Additionally, a 1.0% agarose gel was run to check the size and integrity of the isolated DNA. The extracted genomic DNA was stored at −20°C and subjected to DNA sequencing at Baseclear, Leiden, the Netherlands using the Illumina Sequencing platform and to the Institute for Genome Sciences (IGS), Baltimore, Maryland, USA for PacBio real‐time DNA sequencing.

### *De novo* assembly of *Paenibacillus* sp. AD87 and *Burkholderia* sp. AD24 genomes

From the paired‐end Illumina sequencing platform, an average read length of 101 bp was obtained, and from the PacBio RS platform (Pacific Biosciences, Menlo Park, CA, USA) using the P4‐C2 chemistry, an average read length of 8184 nucleotides for *Paenibacillus* sp. AD87 and 8334 for *Burkholderia* was obtained. The PacBio raw sequences were analysed using SMRT Portal V2.3.0.140936. p.4150482. The sequences were assembled with RS_HGAP_assembly 3 protocol (© Copyright 2010–2014, Pacific Biosciences, Menlo Park, CA, USA) at default settings with estimated genome sizes of 8 MBp for *Burkholderia* and 7 MBp for *Paenibacillus*. The resulting assemblies were subjected to scaffolding using the RS_AHA_scaffolding 1 protocol. The Illumina reads were filtered using Fastq MCF with default settings and aligned against the scaffolds using BWA V0.7.12. The aligned reads were re‐aligned with gatk V3.5.0. The scaffolds of both genomes were corrected using the re‐aligned reads with Pilon (Walker *et al*. 2014). The resulting improved contigs were subjected to another round of scaffolding in SMRT Portal and further corrected. The whole genome assembly properties are shown in Table S1. The final contigs were annotated using a modified version of prokka V1.11 (Seemann, 2014) and interproscan 5.16 55.0 (Jones *et al*., 2014). Both genomes were submitted to the NCBI genome database under accession numbers NCBI Genbank Accession: PRJNA320371 (*Burkholderia* sp. AD24) and LXQN00000000 (*Paenibacillus* sp. AD87).

### Transcriptome analysis

The obtained Illumina reads from the RNA sequencing were filtered using Fastq MCF and aligned against the cDNA sequences of both *Burkholderia* and *Paenibacillus* combined using Bowtie 2 (Langmead and Salzberg, 2012) with the following settings: – no‐mixed – no‐discordant – gbar 1000 – end‐to‐end. Transcript abundance was estimated using rsem V1.1.26 (Li and Dewey, 2011) and differential expression between the treatments was analysed using edger V3.2 (Robinson *et al*., 2010; Zhou *et al*., 2014). Data were filtered with a *P*‐value of 0.001 and a FDR value < 0.05. The results from the transcriptome analysis were confirmed by performing qRT‐PCRs of selected genes (Table S5).

### *In silico* analysis of secondary metabolite gene clusters

For *in silico* analysis of secondary metabolite gene clusters, whole genome sequences of *Burkholderia* sp. AD24 and *Paenibacillus* sp. AD87 were submitted to the antiSMASH website (http://antismash.secondarymetabolites.org/) (Medema *et al*., 2011).

### COG annotation

COG annotations were determined for both *Burkholderia* and *Paenibacillus* using custom scripts based on a modified version of methods described by Snel *et al*. (2002) with COG annotations from Galperin *et al*. (2015).

### Reverse transcription and quantitative real‐time PCR

To confirm the RNA sequencing results, the gene expression of two gene clusters related to secondary metabolite production (a Pederin‐like compound in *Paenibacillus* and a polyketide synthase in *Burkholderia*) was targeted and quantified via quantitative real‐time PCR. For this purpose, the previously extracted RNA was used to synthesize first strand cDNA using the SuperScript^®^ VILO™ MasterMix (Invitrogen, cat#11755050). The concentration and quality of the cDNA was determined using a NanoDrop™ spectrophotometer, and additionally all cDNA samples were run on a 1.0% agarose gel. The selected gene cluster in *Burkholderia* was targeted with the primer combinations bAD24_10391_IG_F and bAD24_10391_IG_R amplifying 322 bp from gene bAD24_10391 encoding SnoaL‐like polyketide cyclase family protein. For normalization, the primers RecA_bAD24_1_F with RecA_bAD24_1_R amplifying 230 bp from gene recA encoding DNA recombination and repair protein were used. The gene clusters in *Paenibacillus* were targeted with the primer combination gpAD87_304F and gpAD87_304R amplifying 592 bp from gene encoding a dimodular nonribosomal peptide synthase. For normalization, the two primers BacF (Garbeva *et al*., 2003) with Eub518R (Fierer *et al*., 2005) amplifying 440 bp from the 16S rRNA gene and primers RecA_gpAD87_3_F and RecA_gpAD87_3_R amplifying 259 bp from gene recA encoding DNA recombination and repair protein were used. From each treatment, 5 μl cDNA was subjected to quantitative RT‐ PCR using QuantiNova SYBR Green I PCR master mix (Qiagen, Cat# 208056) or iTaq™ Universal SYBR^®^ green (BIORAD Cat# 1725122, Bio‐Rad Laboratories B.V., Veenendaal, The Netherlands). For quantification, two‐step quantitative RT‐PCRs were performed on a Qiagen Research Rotor‐Gene Q thermal cycler (Qiagen, Cat# 9001550), with the following settings: initial cycle 95°C for 2 min, followed by 40 cycles of 95°C for 5 s (denaturation) and 60°C for 10 s (combined annealing/extension). All analyses were performed in triplicate. Five standard curves were established to calculate the expression (CT‐value). Gene expression was calculated relative to recA gene of *Burkholderia* and to the recA and 16S rDNA gene of *Paenibacillus* using the 2‐ΔΔCt method (Livak and Schmittgen, 2001). All used primers for qPCR analysis are listed in Table S6.

### Trapping of volatile organic compounds

For analysis of volatile organic compounds, glass Petri dishes were used (Garbeva *et al*., 2014a). The Petri dishes were closed by a lid with an outlet connected to a steel trap containing 150 mg Tenax TA and 150 mg Carbopack B (Markes International, Llantrisant, UK). The Tenax steel traps were collected after 72 h of incubation and stored at 4°C until GC‐Q‐TOF analysis. As controls glass Petri dishes containing 1/10th TSBA media without inoculated bacteria were used.

### GC‐Q‐TOF analysis

The trapped volatile organic compounds were desorbed from the traps using an automated thermos desorption unit (Unity TD‐100, Markes International) at 210°C for 12 min (He flow 50 ml min^−1^) and trapped on a cold trap at −10°C. The trapped volatiles were introduced into the GC‐QTOF (model Agilent 7890B GC and the Agilent 7200A QTOF, Santa Clara, CA, USA) by heating the cold trap for 12 min to 250°C. Split ratio was set to 1:10, and the column used was a 30 × 0.25 mm ID RXI‐5MS, film thickness 0.25 μm (Restek 13424‐6850, Bellefonte, PA, USA). Temperature program used was as follows: 39°C for 2 min, from 39°C to 95°C at 3.5°C min^−1^, then to 165°C at 4°C min^−1^, to 280°C at 15°C min^−1^ and finally to 320°C at 30°C min^−1^, hold 7 min. A constant gas flow of 1.2 ml min^−1^ was used. Volatile organic compounds were ionized in EI mode at eV. Mass spectra were acquired in full‐scan mode (30–400 U @ 5 scans s^−1^). Mass spectra were extracted with masshunter qualitative analysis Software V B.06.00 Build 6.0.633.0 (Agilent Technologies, Santa Clara, CA, USA) and exported as mzXML files for further processing in mzmine V2.14.2. The files were imported to mzmine V2.14.2 (Copyright © 2005–2012 MZmine Development Team; Pluskal *et al*., 2010), and compounds were identified via their mass spectra using deconvolution function in combination with two mass spectral libraries: NIST 2014 V2.20 (National Institute of Standards and Technology, USA http://www.nist.gov) and Wiley 7th edition spectral libraries and by their linear retention indexes (LRI). The LRI values were calculated using amdis 2.72 (National Institute of Standards and Technology, USA). After deconvolution and mass identification peak lists containing the mass features of each treatment were exported in csv file format and uploaded to metaboanalyst V3.0 (www.metaboanalyst.ca).

### Extraction of secondary metabolites

Secondary metabolites were extracted from the same samples used for bacterial enumeration and RNA isolation. The agar was cut into pieces of about 2 cm^2^ and transferred to 50 ml Greiner tubes. After sample collection, the tubes were immediately stored at −80°C. All samples were afterwards transferred to a freeze drier (Labconco Freezone 12 with Labconco Clear Drying Chamber nr.7867000) and freeze‐dried for ~48 h. Subsequently, samples were transferred to a ceramic mortar and a volume of ~100 ml liquid N_2_ was added and the agar pieces were crushed using a pestle. The resulting powder was transferred to 1.2‐mL cryo tubes (Nalgene^®^ cryogenic tubes, Sigma‐Aldrich, Cat# V4757, Sigma Aldrich N.V., Zwijndrecht, The Netherlands). A amount of ~125 mg agar powder was supplemented with 75% methanol + 0.1% formic acid (Merck Methanol, Cat# 106 009, Merck formic acid Cat# 100 253) after solvent addition tubes were vortexed for 30 s. The tubes were afterwards transferred to a sonicator (Bransonic 2510, Branson Ultrasonics B.V., Eemnes, The Netherlands) and sonicated for 30 min. After sonication, the tubes were centrifuged @ 5500 rpm for 20 min and the resulting supernatant was transferred to 1.2 ml tubes and stored at −20°C.

### UHPLC‐ESI‐MS analysis of the extracted secondary metabolites

One microlitre of sample (1 μl) was analysed on a UHPLC system of the Ultimate 3000 series RSLC (Dionex, Dionex Softron GmbH, Germering, Germany) connected to a Q Exactive Hybrid Quadrupole‐Orbitrap mass spectrometer (Thermo Fisher Scientific, Waltham, MA, USA). Separation in the UHPLC system was achieved on an Acclaim C18 column (150 × 2.1 mm, 2.2 μm, Dionex) with a flow rate of 300 μl min^−1^ in a binary solvent system of water and acetonitrile (Merck, hypergrade for LC‐MS), both containing 0.1% (v/v) formic acid (eluent additive for LC‐MS, Sigma‐Aldrich). ESI source parameters were set to 3 kV for spray voltage at a sheath gas flow of 35 and Aux gas flow of 7 l h^−1^. The voltage in the transfer capillary was set to 35 V at a capillary temperature of 325°C. The samples were measured in positive ion mode in the mass range of *m/z* 100–1200 using 140 000 m/Δm resolving power in the Orbitrap mass analyser. Data were interpreted using xcalibur software (Thermo Fisher Scientific). For statistical analysis, the raw spectra were converted to mzXML format using the MS Convert feature of proteowizard 3.0.3750. Subsequently, data processing was carried out with r studio 0.96.316 using the Bioconductor XCMS package. The resulting list (mz, mzmin, mzmax, rt, rtmin, rtmax and peak intensities/areas) was saved in csv file format and uploaded to metaboanalyst V3.0 (www.metaboanalyst.ca).

### Ambient mass spectrometry analysis (LAESI‐MS)

For LAESI‐MS analysis, a single colony of each bacterial isolate was picked and inoculated in 20 ml 1/10th TSB and grown overnight at 24°C, 220 rpm. An inoculation mix of each treatment (*Burkholderia*,* Paenibacillus* and the mix of both) was prepared by diluting the bacterial isolates in 1 ml 10 mM phosphate buffer (pH 6.5). A volume of 5 μl of monocultures or mixture was spotted in duplicates on 1/10th TSBA plates at a distance of approximately 6 cm from each other, resulting in two single colonies per Petri dish. At 24, 48 and 72 h of incubation, bacterial colonies were cut out of the agar (size approximately 1 cm^2^) and subjected to LAESI‐MS measurement. The LAESI‐MS analysis was carried out on a Protea Biosciences DP‐1000 LAESI system (Protea Bioscience, Morgantown, WV, USA) that was coupled to a Waters model Synapt G2S (Waters Corporation, Milford, MA, USA) mass spectrometer. The LAESI system was equipped with a 2940‐nm mid‐infrared laser yielding a spot size of 100 μm. The laser was set to fire 10 times per *x*‐*y* location at a frequency of 10 Hz and 100% output energy. The system was set to shoot at 100 locations per bacterial colony (grid of 10 × 10 positions). A syringe pump was delivering the solvent methanol/water/formic acid (50:50:0.1% v/v) at 2 ml min^−1^ to a PicoTip (5 cm × 100 μm diameter) stainless steel nanospray emitter operating in positive ion mode at 3800 V. The LAESI was operated using laesi desktop software V2.0.1.3 (Protea Biosciences). The time‐of‐flight (TOF) mass analyser of the Synapt G2S was operated in the V‐reflectron mode at a mass resolution of 18.000–20.000. The source temperature was 150°C, and the sampling cone voltage was 30 V. The positive ions were acquired in a mass range of 50–1200 *m/z*. The MS data were lock mass corrected post data acquisition using leucine encephalin (C_25_H_37_N_5_O_7_ *m/z *=* *556.2771), which was added as internal standard. Ions of potential interest for the generation of accurate ion maps (±1 ppm) were identified via background subtraction using MassLynx software (Waters Corporation, Milford, MA, USA). Ion maps were created using protea plot V2.0.1.3 (Protea Biosciences).

### Antibacterial assays

The extracted secondary metabolite was applied in agar disc‐diffusion tests in 9‐cm Petri dishes (Balouiri *et al*., 2016). Single colonies of *E. coli* WA321 and *S. aureus* 533R4 were picked from plate and grown overnight in liquid LB broth at 37°C, 220 rpm. Fresh LB‐agar (1.5% Merck Agar) was prepared and cooled down to ~45°C, and the target organisms were added to a final OD_600_ of 0.002 corresponding to approximately 6 × 10^5^ CFU ml^−1^ (*E. coli* WA321) or 4 × 10^5^ CFU ml^−1^ (*S. aureus* 533R4). To each plate, 15 ml of the seeded agar was added and after solidification, six filter papers with a diameter of ~5.5 mm (Whatman™, Cat# 1003‐150, 6 μm pore size, Fisher Scientific B.V., Landsmeer, The Netherlands) were placed on the top of the agar surface. A volume of 5 μl of each extract was added onto the filter papers. As control a volume of 5 μl of the solvent (75% methanol) was added. As positive control 5 μl of appropriate antibiotic (ampicillin 100 mg ml^−1^ for *E. coli* WA321 or tetracycline 15 mg ml^−1^ for *S. aureus* 533R4) was used. As negative control filter paper with no supplemented antibiotics or secondary metabolite extracts were applied. The plates were incubated overnight at 37°C. The next day plates were examined for visible zones of inhibition (ZOI) and digital photographs were taken. The digital images were analysed using the axio vision v4.8 imaging software (Carl Zeiss Carl Zeiss B.V., Breda, the Netherlands) for surface‐area determination (in pixel^2^). All treatments were performed in six replicates.

### In vitro test of biological activity of 2,5‐bis(1‐methylethyl)‐pyrazine on *Burkholderia* sp. AD24 and *Paenibacillus* sp. AD87

For the determination of *in vitro* biological activity of 2,5‐bis(1‐methylethyl)‐pyrazine on the two used soil bacteria absorbance measurements combined with CFU counting were performed in 96‐well plates (Greiner Bio‐One, Cat# 655180, Greiner Bio‐One B.V., Alphen a/d Rijn, the Netherlands). To this end, single colonies of either *Burkholderia* or *Paenibacillus* were picked and grown in 20 ml 1/10th TSB overnight at 24°C, 220 rpm.. Three concentrations of 2,5‐bis(1‐methylethyl)‐pyrazine were tested (10% v/v = 10 μl (=9.2 mg), 5% v/v = 5 μl (=4.6 mg) and 2% v/v (=1.84 mg). The pyrazine compound was added to 100 μl 1/10th TSB containing the soil bacterial isolates with an optical density (OD_600_) of 0.05. As positive assay control 5 μl of 15 mg ml^−1^ tetracycline (=75 μg) (Sigma‐Aldrich product # T‐7660) and 10 μl of CHCl_3_ was applied as solvent control (Merck Chloroform, Cat# 102 445). The growth rates of the soil bacteria were monitored in the presence of 2,5‐bis(1‐methylethyl)‐pyrazine compared to the controls (soil bacteria in the absence of 2,5‐bis(1‐methylethyl)‐pyrazine) using a BioTek Synergy™ HT Multi‐Mode Microplate Reader (Beun de Ronde Life Sciences, Beun de Ronde B.V., Abcoude, the Netherlands). The assay was performed at 24°C for 24 h. All measurements were performed in three replicates and normalized against the mean absorbance values of the culture media. After 24 h of incubation, a sample of 100 μl of each well was taken and added to 900 μl of 10 mM phosphate buffer (pH 6.5). Dilution were prepared in triplicates and plated in three replicates on 1/10th TSBA plates. The plates were incubated for 3 days at 24°C, and CFU enumeration was carried out on an aCOlyte Colony Counter as described above.

### Bioassays to test 2,5‐bis(1‐methylethyl)‐pyrazine

The effect of pure 2,5‐bis(1‐methylethyl)‐pyrazine was tested on *E. coli* WA321, *S. aureus* 533R4, *R. solani* AG2.2 IIIB, *F. culmorum* PV and *C. albicans* BSMY 212 (Schmidt, 1996; Garbeva *et al*., 2014a; Tyc *et al*., 2014). The assays were performed in 12‐well plates (Greiner Bio‐One, Cat# 665180). Stock solutions of 50 μl 2,5‐bis(1‐methylethyl)‐pyrazine (Sigma‐Aldrich, Cas# 24294‐83‐5) were prepared. The model organisms *E. coli* WA321, *S. aureus* 533R4 and *C. albicans* BSMY212 were grown overnight either in liquid LB or liquid YEPD broth at 37°C, 220 rpm. Fresh LB‐ and YEPD agar (1.5% Merck Agar) was prepared and cooled down to ~45°C, and the target organisms were added to the liquid agar at a final OD_600_ of 0.002. A volume of 1 ml liquid agar seeded with the test organisms was added to each well. For the test on mycelial growth, fresh PD‐agar (1.0% Merck Agar) was prepared and a volume of 1 ml was added to each well. The fungi were added by placing a 5 mm diameter fungal plug of *R. solani* AG2.2 IIIB, *F. culmorum* PV at the top edge of each well. A filter paper with a diameter of ~5.5 mm (Whatman™, Cat# 1003‐150, 6 μm pore size) was placed on the agar at the lower edge of each compartment. A droplet of 2 μl 2,5‐bis(1‐methylethyl)‐pyrazine (=1.84 mg) was added onto each filter paper. As controls 2 μl of the solvent (CHCl_3_) was applied (Merck Chloroform, Cat# 102 445). The plates were incubated overnight at 37°C (*E. coli*,* S. aureus* & *C. albicans*) or at 24°C for 4 days (*R. solani, F. culmorum*). After incubation, plates were examined for visible zones of inhibition (ZOI) or inhibition of fungal growth (mycelial extension) and digital photographs were taken. The digital images were analysed using axio vision v4.8 imaging software (Carl Zeiss B.V., Breda, the Netherlands).

### Test for additive effects between secondary metabolite extracts and 2,5‐bis(1‐methylethyl)‐pyrazine

Agar diffusion tests with secondary metabolite extracts of the interaction *Burkholderia* with *Paenibacillus* in combination with the pure volatile compound 2,5‐bis(1‐methylethyl)‐pyrazine were performed. The assays were carried out in 12‐well plates (Greiner bio‐one, Cat# 665180). A stock solution of 25 μl pure 2,5‐bis(1‐methylethyl)‐pyrazine was prepared in a 1.7 ml tube. The model organisms *E. coli* WA321 and *S. aureus* 533R4 were grown overnight in liquid LB broth at 37°C, 220 rpm. Fresh LB‐agar (1.5% Merck Agar) was prepared and cooled down to ~45°C, and the target organisms were added to the liquid agar at a final OD_600_ of 0.002. A volume of 1 ml liquid agar seeded with the test organisms was added to each well. A filter paper with a diameter of ~5.5 mm (Whatman™ filter paper Cat# 1003‐150, 6 μm pore size) was placed in the middle of the solidified agar in each compartment. A droplet of 7 μl containing 2 μl 2,5‐bis(1‐methylethyl)‐pyrazine (=1.84 mg) and 5 μl of the secondary metabolite extracts was added onto the filter paper. As controls filter papers were supplemented with 7 μl of the solvents (5 μl Merck Methanol, Cat# 106 009 and 2 μl Merck Chloroform, Cat# 102 445). As positive assay control 2 μl of antibiotics (ampicillin 100 mg ml^−1^ for *E. coli* WA321 or tetracycline 15 mg ml^−1^ for *S. aureus* 533R4) was applied. As negative assay control filter paper with no supplemented antibiotics or secondary metabolite extracts were applied. The plates were incubated overnight at 37°C. After overnight incubation, plates were examined for visible zones of inhibition (ZOI) and digital photographs were taken. The digital images were analysed using axio vision v4.8 imaging software (Carl Zeiss Imaging Solutions GmbH) for surface‐area determination (in pixel^2^). All treatments were performed in six replicates.

### Statistical analysis

Statistical analyses on cell counts, zone of inhibition size (ZOI) and fungal growth (mycelial extension) were performed with ibm spss Statistics 24 (IBM, Somers, NY, USA) using one‐way ANOVA with *post hoc* TUKEY (HSD‐test) between the treatments. Significant differences between the treatments and the controls are indicated by different letters or asterisks (*P *<* *0.05). Statistical analysis on volatile and soluble metabolites data was performed using metaboanalyst V3.0, www.metaboanalyst.ca (Xia *et al*., 2015). Prior to statistical analysis, data normalization was performed via log‐transformation. To identify significant abundant masses, one‐way ANOVA with post hoc TUKEY test was performed between the data sets. To identify important mass features, PLSD‐analysis was performed. Masses were considered to be statistical relevant if FDR values were ≤ 0.05. Statistical analysis on gene expression data was carried out using edger package V3.2, and the data was filtered with a *P*‐value of < 0.001 and a FDR value of < 0.05.

## Conflict of interest

The authors declare that the research was conducted in the absence of any commercial or financial relationships that could be construed as a potential conflict of interest.

## Supporting information


**Fig. S1.** Normalized concentration of the unknown Pederin like compound with a mass of 504.316 (M+H^+^).
**Fig. S2.** Representation of GC/MS chromatograms of (**A)** *Burkholderia* sp. AD24 monoculture (top) (**B)** *Paenibacillus* sp. AD87 monoculture (middle) and (**C)** interaction of both bacteria (bottom). The compound 2,5‐bis(1‐methylethyl)‐pyrazine (RT 19.7, m/z = 164.247) was detected in a higher concentration in samples of the pairwise combination of *Burkholderia* sp. AD24 with *Paenibacillus* sp. AD87.
**Fig. S3.** Effect of 2,5‐bis(1‐methylethyl)‐pyrazine on growth of target organisms.
**Fig. S4.** Effect of the secondary metabolite extracts and the interaction extracts and the combination of the extracts with 2,5‐bis(1‐methylethyl)‐pyrazine on growth of *S. aureus*.
**Table S1.** Genome assembly results for *Burkholderia* sp. AD24 and *Paenibacillus* sp. AD87.
**Table S2.** List of the differentially up‐ and down regulated genes (FDR ≥0.05, FC ± >2) in *Burkholderia* sp. AD24 during interaction with *Paenibacillus* sp. AD87 over the time points t = 48 h to t = 72 h.
**Table S3.** List of the top differentially up‐ and down regulated genes (FDR ≥0.05, FC ± >3) in *Paenibacillus* sp. AD87 during interaction with *Burkholderia* sp. AD24 over the time points t = 48 h to t = 72 h.
**Table S4.** Bacterial, fungal and oomycetal organisms used in this study.
**Table S5.** Outcome of the qRT‐PCR analysis and comparison to the outcome of the RNA‐seq analysis.
**Table S6.** Primers used for quantitative real‐time PCRs.
