# Supplementary material for: Exploring bacterial interspecific interactions for discovery of novel antimicrobial compounds
Source: Microb Biotechnol. 2017 May 29;10(4):910–25. doi: 10.1111/1751-7915.12735 (PMC5481530; doi:10.1111/1751-7915.12735)
Supplement: Supplementary file 1 — Fig. S1. Normalized concentration of the unknown Pederin like compound with a mass of 504.316 (M+H+). Fig. S2. Representation of GC/MS chromatograms of (A) Burkholderia sp. AD24 monoculture (top) (B) Paenibacillus sp. AD87 monoculture (middle) and (C) interaction of both bacteria (bottom). The compound 2,5‐bis(1‐methylethyl)‐pyrazine (RT 19.7, m/z = 164.247) was detected in a higher concentration in samples of the pairwise combination of Burkholderia sp. AD24 with Paenibacillus sp. AD87. Fig. S3. Effect of 2,5‐bis(1‐methylethyl)‐pyrazine on growth of target organisms. Fig. S4. Effect of the secondary metabolite extracts and the interaction extracts and the combination of the extracts with 2,5‐bis(1‐methylethyl)‐pyrazine on growth of S. aureus. Table S1. Genome assembly results for Burkholderia sp. AD24 and Paenibacillus sp. AD87. Table S2. List of the differentially up‐ and down regulated genes (FDR ≥0.05, FC ± >2) in Burkholderia sp. AD24 during interaction with Paenibacillus sp. AD87 over the time points t = 48 h to t = 72 h. Table S3. List of the top differentially up‐ and down regulated genes (FDR ≥0.05, FC ± >3) in Paenibacillus sp. AD87 during interaction with Burkholderia sp. AD24 over the time points t = 48 h to t = 72 h. Table S4. Bacterial, fungal and oomycetal organisms used in this study. Table S5. Outcome of the qRT‐PCR analysis and comparison to the outcome of the RNA‐seq analysis. Table S6. Primers used for quantitative real‐time PCRs. [file MBT2-10-910-s001.docx]

**Supporting Information**

**Exploring bacterial inter-specific interactions for discovery of novel antimicrobial compounds**

**Olaf Tyc^1*^, Victor de Jager^1^, Marlies van den Berg^1^, Saskia Gerards^1^, Thierry Janssens^2^, Niels Zaagman^2^, Marco Kai^3^, Ales Svatos^3^, Hans Zweers^1^, Cornelis Hordijk^1^, Harrie Besselink^4^, Wietse de Boer^1,5^ and Paolina Garbeva^1^**

^1^Netherlands Institute of Ecology (NIOO-KNAW), Department of Microbial Ecology, PO BOX 50, 6700 AB Wageningen, Netherlands

^2^MicroLife Solutions B.V., Science Park 406, 1098 XH Amsterdam, Netherlands

^3^Mass Spectrometry Research Group, Max Planck Institute for Chemical Ecology, Hans-Knoell-Str. 8, D- 07745 Jena, Germany

^4^BioDetection Systems B.V., Science Park 406, 1098 XH Amsterdam, Netherlands

^5^Department of Soil Quality, Wageningen University *&* Research Centre (WUR)*,* PO BOX 47, 6700 AA Wageningen, Netherlands

*** Correspondence:**

Olaf Tyc, Netherlands Institute of Ecology (NIOO-KNAW), Department of Microbial Ecology, PO BOX 50, 6700 AB Wageningen, Netherlands, [o.tyc@nioo.knaw.nl](mailto:o.tyc@nioo.knaw.nl)

**Table S1.** Genome assembly results for *Burkholderia* sp. AD24 and *Paenibacillus* sp. AD87.

**Table S2.** List of the differentially up- and down regulated genes (FDR ≥0.05, FC +/- >2) in *Burkholderia* sp. AD24 during interaction with *Paenibacillus* sp. AD87 over the time points t=48h to t=72h.

**Table S3.** List of the top differentially up- and down regulated genes (FDR ≥0.05, FC +/- >3) in *Paenibacillus* sp. AD87 during interaction with *Burkholderia* sp. AD24 over the time points t=48h to t=72h.

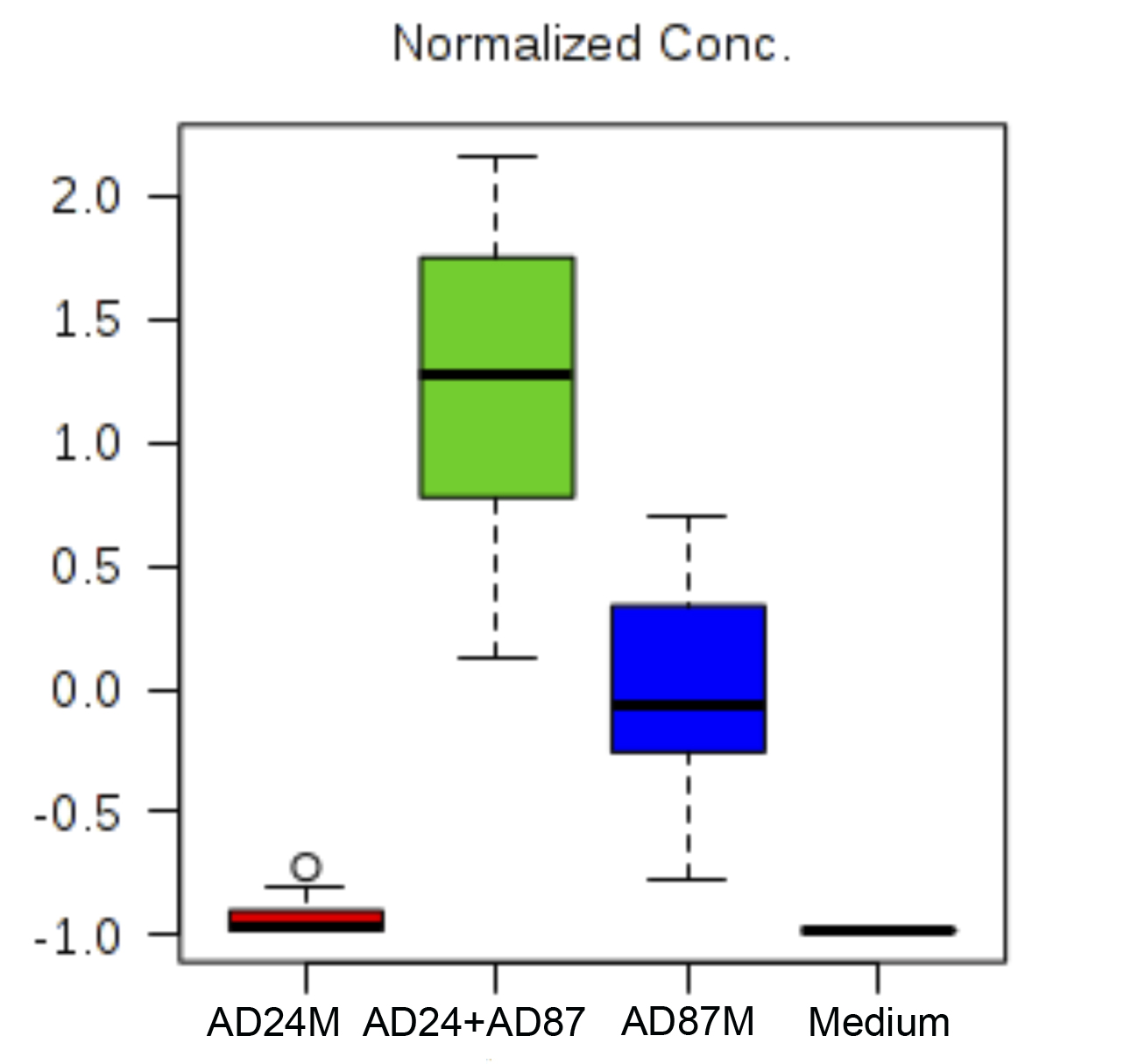


**Fig. S1.** Normalized concentration of the unknown Pederin like compound with a mass of 504.316 (M+H^+^). The compound was detected in a higher concentration during interaction of *Burkholderia* sp. AD24 with *Paenibacillus* sp. AD87. The compound was not detected in samples of *Burkholderia* sp. AD24 monoculture and in the medium control (extracts of 1/10^th^ TSBA).

**
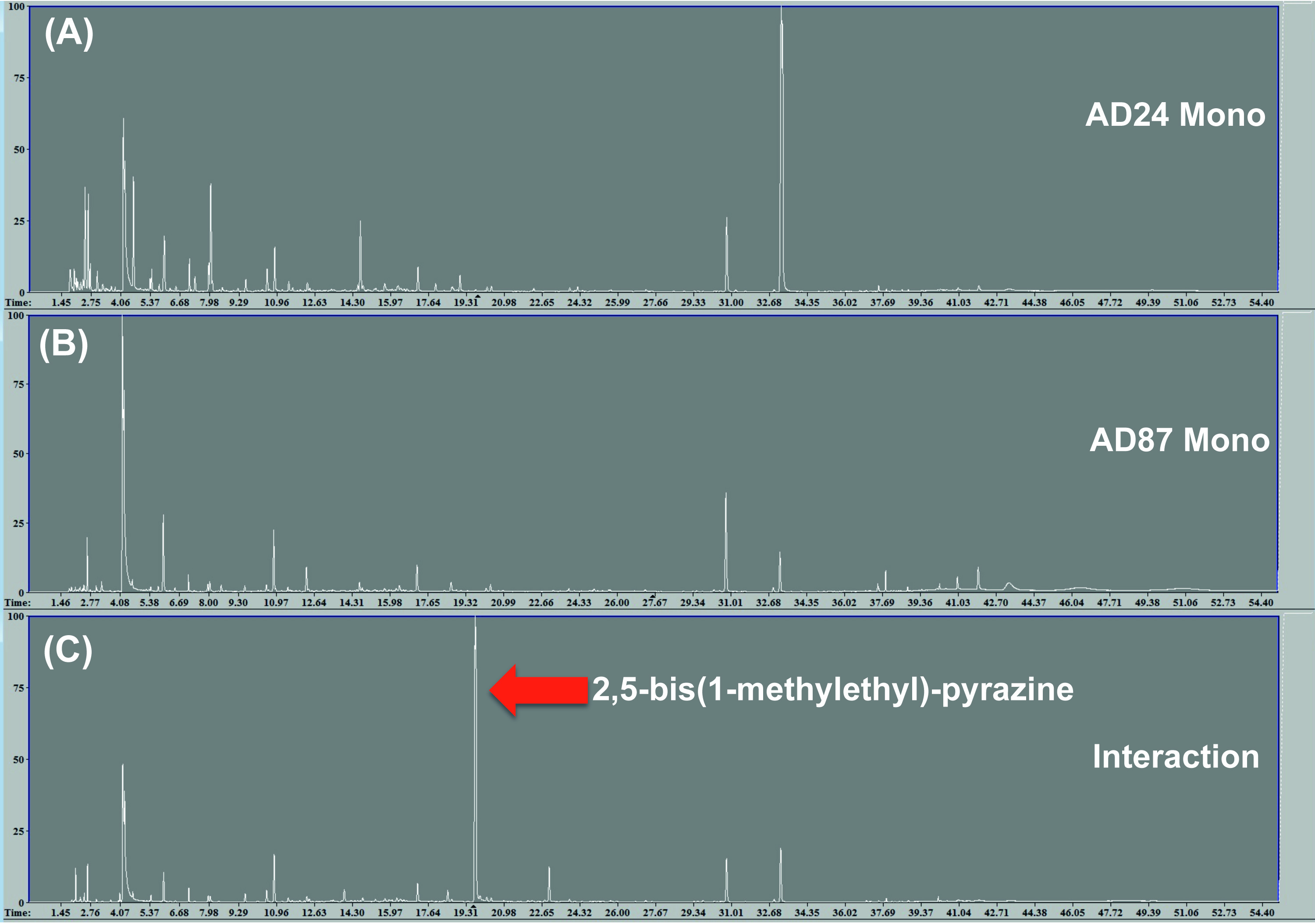
**

**Fig. S2.** Representation of GC/MS chromatograms of (**A)** *Burkholderia* sp. AD24 monoculture (top) (**B)** *Paenibacillus* sp. AD87 monoculture (middle) and (**C)** interaction of both bacteria (bottom). The compound 2,5-bis(1-methylethyl)-pyrazine (RT 19.7, m/z= 164.247) was detected in a higher concentration in samples of the pairwise combination of *Burkholderia* sp. AD24 with *Paenibacillus* sp. AD87.

**
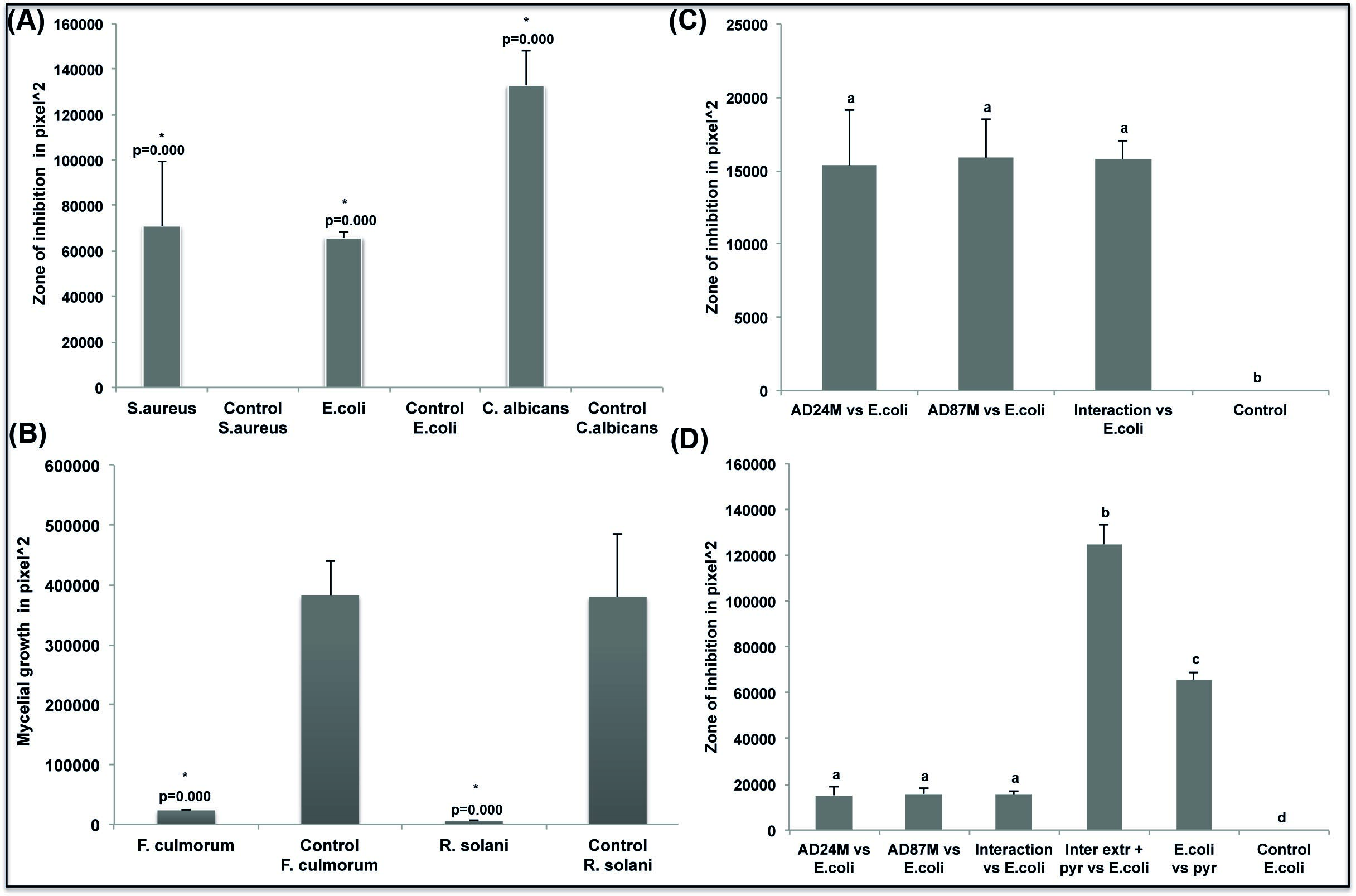
**

**Fig. S3.** Effect of 2,5-bis(1-methylethyl)-pyrazine on growth of target organisms. (**A**) effect on *E. coli* WA321, *S. aureus* 533R4 and *C. albicans* BSMY212 growth. Bars represent the mean sizes of the Zone of inhibition (ZOI) in pixel^2 (**B**) effect of 2,5-bis(1-methylethyl)-pyrazine on mycelial extension of *R. solani* and *F. culmorum.* (**C**) Effect of the secondary metabolite extracts on *E. coli* WA321. (**D**) Effect of the secondary metabolite extracts in combination with 2 µl 2,5-bis(1-methylethyl)-pyrazine revealing a synergistic effect between the secondary metabolite extracts and the pure pyrazine compound. Bars represent the mean sizes of the Zone of inhibition (ZOI) in pixel^2. Error bars are indicating standard deviation (SD) between the replicates. Significant differences between the treatments are indicated by different letters (ONE-WAY ANOVA, post-hoc TUKEY test p<0.05). Bars represent the mean of mycelial extension in pixel^2. Error bars represent standard deviation (SD).

**
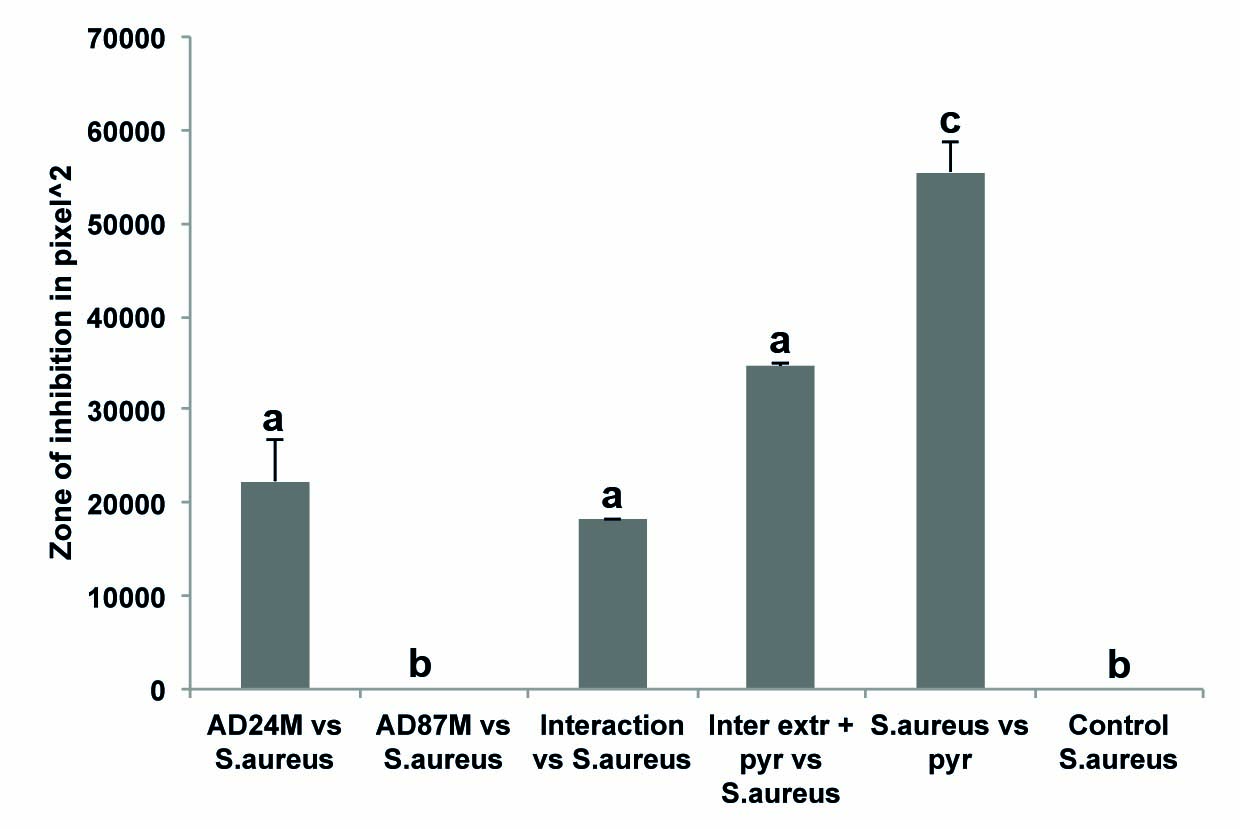
**

**Fig. S4.** Effect of the secondary metabolite extracts and the interaction extracts and the combination of the extracts with 2,5-bis(1-methylethyl)-pyrazine on growth of *S.aureus*. Bars represent the mean sizes of the Zone of inhibition (ZOI) in pixel^2. Error bars are indicating standard deviation (SD) between the replicates. Significant differences between the control and the treatments are indicated by different letters (ONE-WAY ANOVA, post-hoc TUKEY test p<0.05).

**Table S4.** Bacterial, fungal and oomycetal organisms used in this study.

**Table S5.** Outcome of the qRT-PCR analysis and comparison to the outcome of the RNA-seq analysis. The fold change values for the qRT-PCR are analysis are calculated with the ΔΔ-CT method.

**Table S6.** Primers used for quantitative real-time PCRs.
